# ^18^F-Fluorination: Challenge and Opportunity for Organic Chemists

**DOI:** 10.1021/acs.joc.1c01474

**Published:** 2021-08-24

**Authors:** Riya Halder, Tobias Ritter

**Affiliations:** † Max-Planck-Institut für Kohlenforschung, Kaiser-Wilhelm-Platz 1, 45470 Mülheim an der Ruhr, Germany

## Abstract

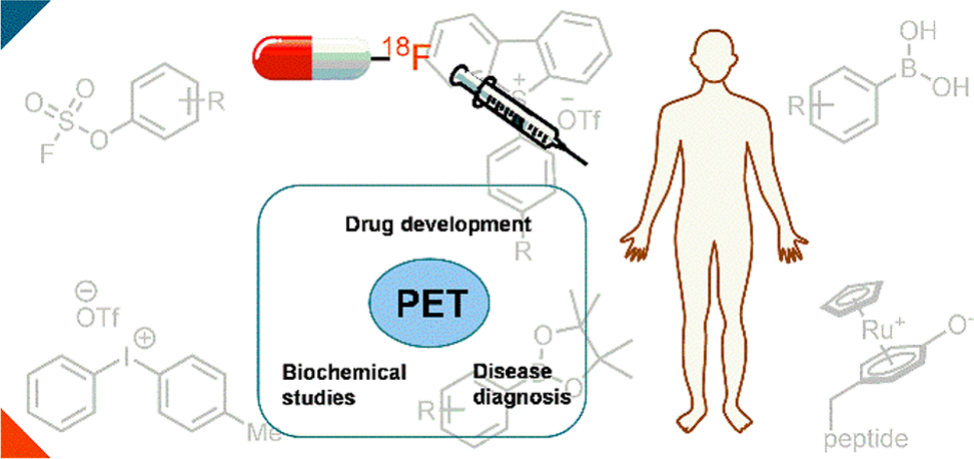

^18^F-fluorination is an important and growing field in organic synthesis that has attracted many chemists in the recent past. Here we present our own, biased perspective with a focus on our own chemistry that evaluates recent advances in the field and provides our opinion on the challenges for the development of new chemistry, so that it may have an impact on imaging. We hope that the manuscript will provide a useful guide to chemists to develop reliable and robust reaction chemistry suitable for radiofluorination to have a real impact on human health.

## Introduction

**Figure 1:**
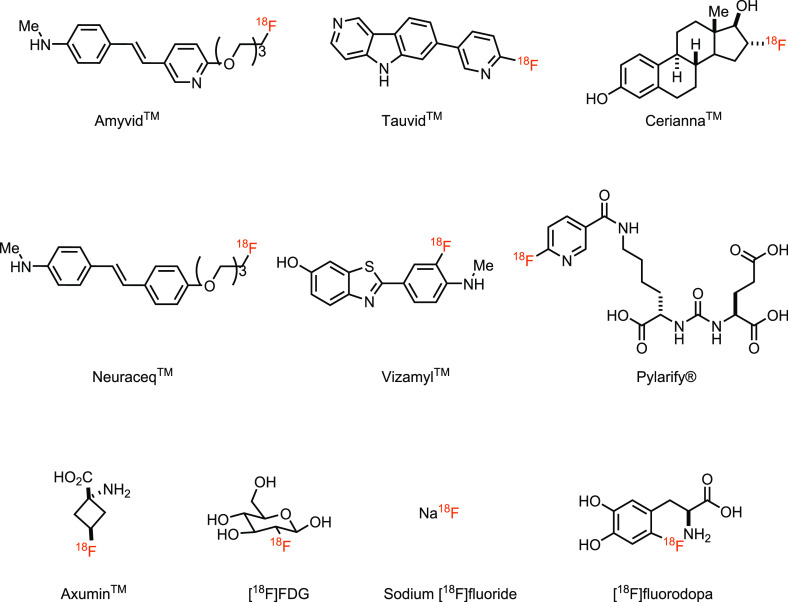
Fluorine-18 containing FDA-approved PET tracers as of August 2021.

Positron-emission-tomography (PET) is a noninvasive imaging method that allows the study of the distribution of radiolabeled compounds in vivo and thereby gives information about physiology such as metabolism, receptor concentration, and transport across cell membranes.^1^ An early diagnosis of diseases by monitoring unusual changes of these processes can improve patient outcomes because they can occur before anatomic changes can be observed.^2^ Various radionuclides have been used to develop PET tracers, and those labeled with fluorine-18 have been used successfully for more than 30 years.^3^ The advantageous nuclear decay properties of fluorine-18, for example, a half-life of 109.7 min (longer than other radionuclides such as carbon-11, nitrogen-13, and oxygen-15), permit its use in multistep synthesis and also shipment of PET tracers from nuclear pharmacies to imaging centers.^4^ Ideally, the radionuclide’s half-life should be long enough for the tracer to achieve an optimal target-to-background ratio while remaining as short as feasible to reduce the patient’s radiation exposure. Moreover, the maximum positron emission energy of fluorine-18 (635 keV) is the smallest among common radionuclides, which results in high image resolution.^5^ As of August 2021, there are 16 FDA-approved PET tracers, out of which 10 contain fluorine-18 (Figure 1).^6,7^ The most common fluorine-18 containing PET tracer is 2-deoxy-2-[^18^F]fluoroglucose or [^18^F]Fluorodeoxyglucose ([^18^F]FDG), a glucose analogue with the hydroxyl substituent in the two-position replaced with its bioisostere fluorine-18, which is extensively used in oncology.^8^

Because future developments in PET will likely contribute to improving human health, and synthetic chemistry is a bottleneck for the development of new PET tracers, we offer here a personal perspective to chemists that critically analyzes selected recent approaches by organic chemists in the field of radiofluorination, with a special emphasis on our own chemistry, explains challenges, and discusses most promising directions we see for chemists to make a real impact in human health. The piece is not a comprehensive review of the field, and we apologize to all our colleagues whose important chemistry is not included due to our own biased selection. We invite more organic chemists to consider making a contribution to this exciting field: The development of fluorination reaction chemistry that can be successfully and reliably translated to radiochemistry has the potential to impact PET tracer development beyond the laboratories of organic chemists and may ultimately contribute to improving human health.

## Discussion

There are two conceptually different approaches with respect to the information that can be obtained from a PET scan.^9^ First, imaging a radiolabeled drug molecule gives information about the drug itself, its biodistribution, or at least the biodistribution of the molecular entity attached to the label if metabolized, and pharmacokinetics in the body. Second, a radiotracer with a known affinity for a molecular target of interest provides data about that target and potentially other molecules, possibly a drug, binding to the same target. Both approaches are valuable but provide different information. If one specific, clearly defined molecule must be labeled, it must contain an atom that can be replaced by a PET-active isotope, meaning that for PET imaging a drug with fluorine-18 the molecule must already contain a fluorine atom that can be replaced to obtain an isotopologue of the original molecule. Otherwise, a different compound would be observed, with different pharmacokinetics (PK) and dynamics (PD). As such, it is important to have robust chemistry available for a large number of functional groups, such as aryl fluorides and trifluoromethylated arenes to name just two examples. The molecular structure is dictated by the pharmaceutical, not the available chemistry. On the other hand, given that only one specific, predefined compound must be labeled, the requirements for the chemistry in terms of practicality and low cost are not as stringent, as scientists are prepared to go through great lengths if the one compound can be accessed at all. For the development of PET tracers, the picture differs in as much that, just as for the development of pharmaceuticals, many different molecules, labeled in this case, must be evaluated to finally arrive at the one with the desired properties. For chemistry to support such a process, reliability, robustness, and straightforward handling is an absolute key, so that radiochemists feel comfortable, and inclined, to use the chemistry. So not every application requires the same chemistry settings. While novel chemistry to access challenging structures can be very helpful for the synthesis of an ^18^F-labeled drug, even if the implementation is somewhat challenging, the go-to method for the evaluation of many different labeled compounds must be robust, reliable, and easy to implement in, ideally, all cases, but is more forgiving in the predefined structure of the molecule, as long as many labeled molecules can be accessed reliably and quickly.

**Figure 2:**
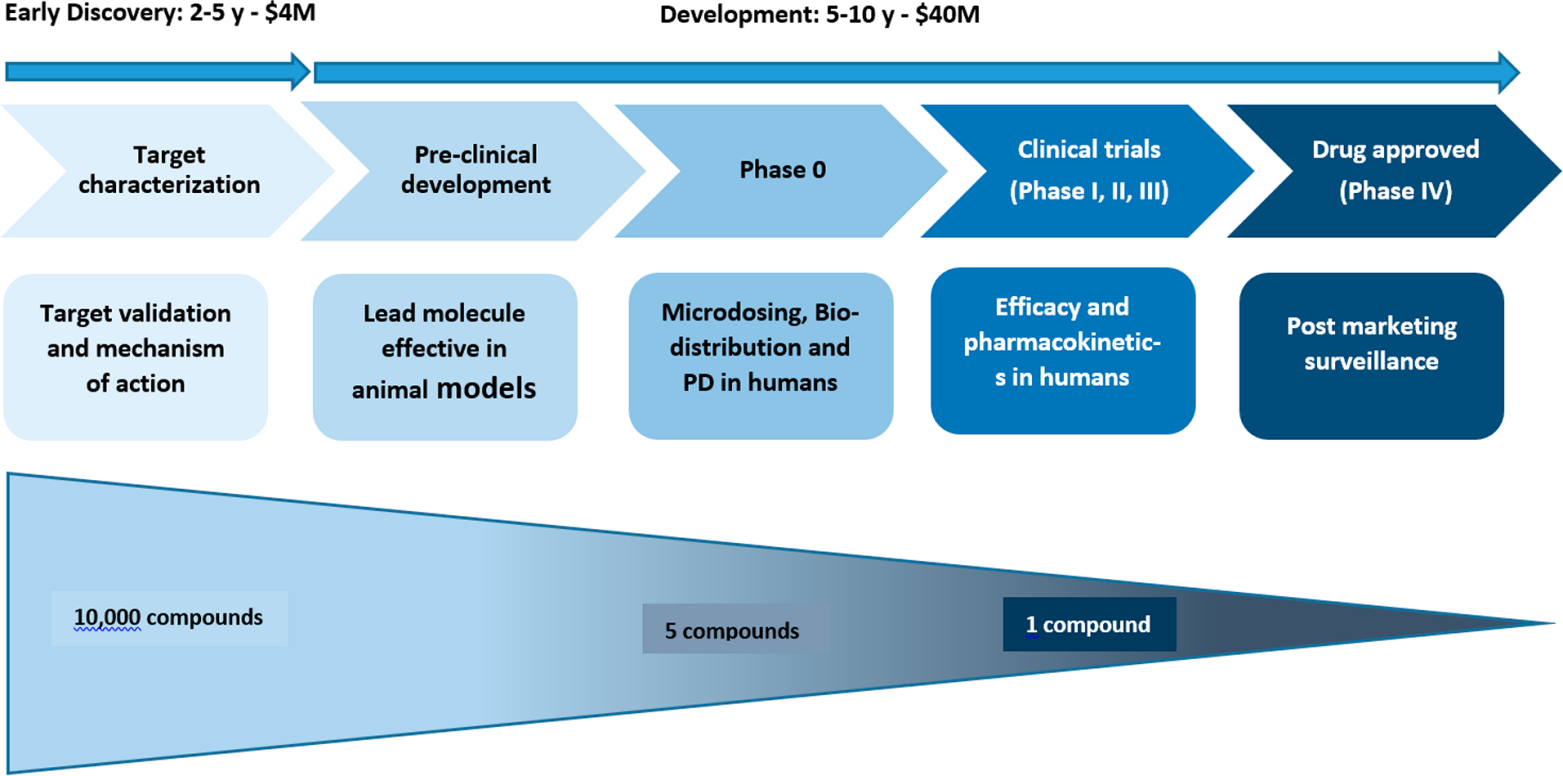
Phases of drug discovery and development.^10,12^

The discovery of a drug begins with the validation of a target, synthesis, and testing of a series of molecules that bind efficiently to the target and, subsequently, optimization of chemical structures that interact with the target to improve the potential as a drug before commencing the actual clinical trials in human (Figure 2).^10^ Thousands of drug candidates are eliminated during this process before the optimal candidate is chosen to move forward into the clinic. Because the costs of clinical drug development increase from phase 1 to 3 so dramatically, early informed decisions in the process become crucial or at least can save hundreds of millions of dollars if molecules that would ultimately fail could be eliminated at the early stages of the development. In a surveying review of several drug candidates, a group of Pfizer scientists highlighted the importance of PET tracers in drug development by stating that “The highest level of confidence and direct evidence at the site of action that required levels of target binding were being achieved is most probably obtained from PKPD [pharmacokinetic/pharmacodynamics] studies of in vivo occupancy measurements with positron emission tomography (PET) or radiolabeled ligands”.^11^ Therefore, a higher throughput assessment of potential tracers than currently possible would increase the rate of discovery. More reliable, general, and operationally simple chemistry would contribute to a broader use of PET for early compound elimination.

Conventionally, fluorine-18 is most commonly introduced to a molecule via nucleophilic substitution (S_N_2 or S_N_Ar) due to the operational simplicity of these reactions. Although the potential of radiotracers in drug discovery has contributed to the development of new and advanced fluorination methods with fluorine-19, the translation to fluorine-18 radiolabeling has been sluggish. A major obstacle in transitioning from fluorine-19 to fluorine-18 chemistry is the requirement of the reaction to be completed quickly, ideally within 30 min or less, due to the half-life of fluorine-18. Another challenge is the low concentration of fluorine-18, which is always the limiting reagent and often only present in nanomole or picomole quantities, in the presence of large excess (millimolar concentration) of the other reaction component. This stoichiometry commonly differs from ^19^F-fluorination reactions by several orders of magnitude. Consequently, modern fluorination reactions, even if useful for fluorine-19 chemistry, may not be readily translated smoothly to radiochemistry. The use of modern techniques such as the radiosynthesis in microfluidic devices can address these shortcomings of conventional batch processes because they allow reactions with minute quantities of solvent and other reagents.^13−15^ Special chips for radiosynthesis have already successfully impacted the synthesis of fluorine-18 labeled molecules, but organic chemists have used such techniques relatively little for the development of new chemical reactions. In this area, we see a potential growth as reaction condition optimization is often key in organic chemistry, and microfluidic devices can provide for a platform with the opportunity to address the stoichiometry aspect in a disruptive fashion as compared to what has been possible in the batch processes typically used so far.

Modern fluorination reactions use nucleophilic, electrophilic, or radical sources of fluorine, with substantial demonstrated success over the last 15 years or so. We entered the field by attempting to solve the challenge of late-stage functionalization/fluorination^16^ for the construction of carbon–fluorine bonds in the presence of a variety of other functional groups, which was an unsolved challenge at the time. All our initial attempts were based on electrophilic fluorination reactions,^17−20^ which is an acceptable approach for the synthesis of high-value compounds although electrophilic fluorinating reagents are more expensive than fluoride. However, the story is more complex when talking about fluorine-18 because electrophilic ^18^F-fluorinating reagents are not as readily accessible. For example, no-carrier-added (n.c.a., without exogenous addition of the PET-inactive fluorine-19) nucleophilic [^18^F]fluoride is produced as an aqueous solution by a cyclotron and is much more practical to make and handle as compared to carrier-added (c.a.) highly reactive [^18^F]F_2_ gas. While [^18^F]fluoride is made through proton bombardment of heavy water, [^18^F]F_2_ gas must be made from ^19^F/^18^F isotopic exchange between carrier F_2_ gas and Me[^18^F]F by application of high voltage electrical discharge,^21^ which is more cumbersome and not available at all cyclotrons.

**Scheme 1:**
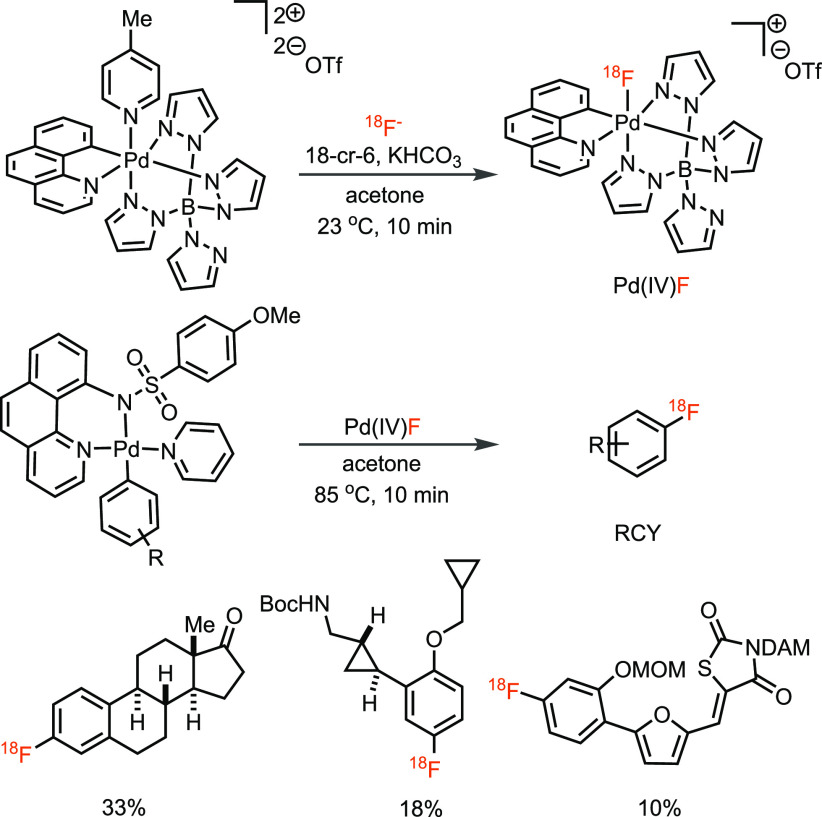
^18^F-Fluorination of Pd–Aryl Complex with Electrophilic Fluorinating Reagent Pd(IV)F

Because many modern fluorination reactions used electrophilic fluorinating reagents, we attempted to synthesize an electrophilic fluorinating reagent with fluorine-18 from fluoride, which is chemically a challenge, as fluorine is the most electronegative element. In 2011, our group reported the successful synthesis of such a reagent.^22^ It involves the binding of [^18^F]fluoride to a cationic Pd(IV) complex to generate a palladium(IV)–[^18^F]fluoride complex that can behave as an electrophilic fluorinating reagent (Scheme 1). In combination with another arylpalladium(II) complex (synthesized by transmetalation from an arylboronic acid derivative), we were able to make fluorine-18 labeled molecules by late-stage fluorination through an oxidative fluorine transfer to give an arylpalladium(IV)–[^18^F]fluoride complex that undergoes C–F reductive elimination to afford ^18^F-labeled aryl fluorides that were not readily accessible with other methods at the time. The conceptual advance was the generation of an electrophilic reagent directly from fluoride, but the operational complexity of the transformation, two palladium complexes, moisture-sensitive reagents, and the requirement for the stoichiometric synthesis of organometallic reagents made this approach, at least from the PET practitioners’ point of view, too challenging to execute, as PET centers around the world would not adopt the involved process.

**Scheme 2:**
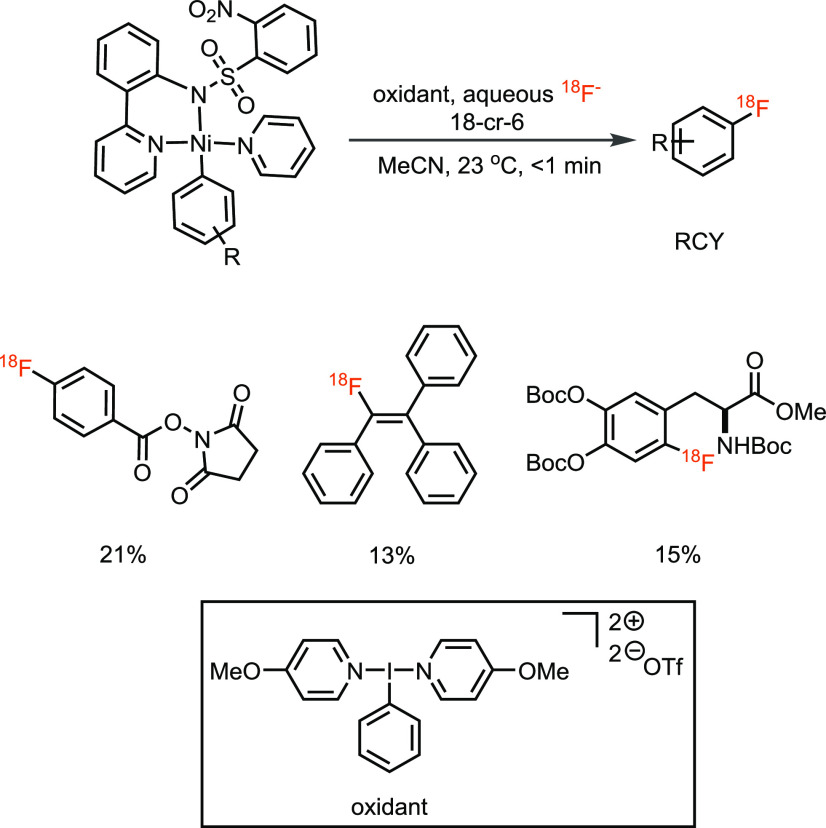
Oxidative ^18^F-Fluorination of Ni–Aryl Complex

The subsequent attempt to facilitate the process by now using only one organometallic, as opposed to two, with nickel complexes resulted in significant but insufficient improvement. One-step radiofluorination of arylnickel(II) complexes (synthesized by oxidative addition of an aryl halide to a Ni(0) precursor) using aqueous [^18^F]fluoride and a dicationic hypervalent iodine-based oxidant proceeds quickly (Scheme 2).^23^ Unlike the palladium-mediated procedure, the one-pot method involved only the nickel aryl complex, fluoride, and oxidant and eliminates the need to prepare a separate electrophilic fluorinating reagent from [^18^F]fluoride. The stability of the nickel aryl complexes allows use in the synthesis of PET tracers, however, the hypervalent iodine oxidant used in the transformation displays limited stability, replacement of which with a suitable, stable oxidant would be a useful advance.

Although not necessarily a deal-breaker, we received criticism about the amount of transition metal used in our procedures. While the use of stoichiometric nickel or even palladium is not an issue from a cost perspective because fluoride-18 is significantly more expensive on the nanomolar scales the reactions are performed at, trace metals must be separated postsynthesis before reformulation as a requirement for injection. While such a step is possible, sometimes even practical, it is an additional process that reduces yield.

**Scheme 3:**
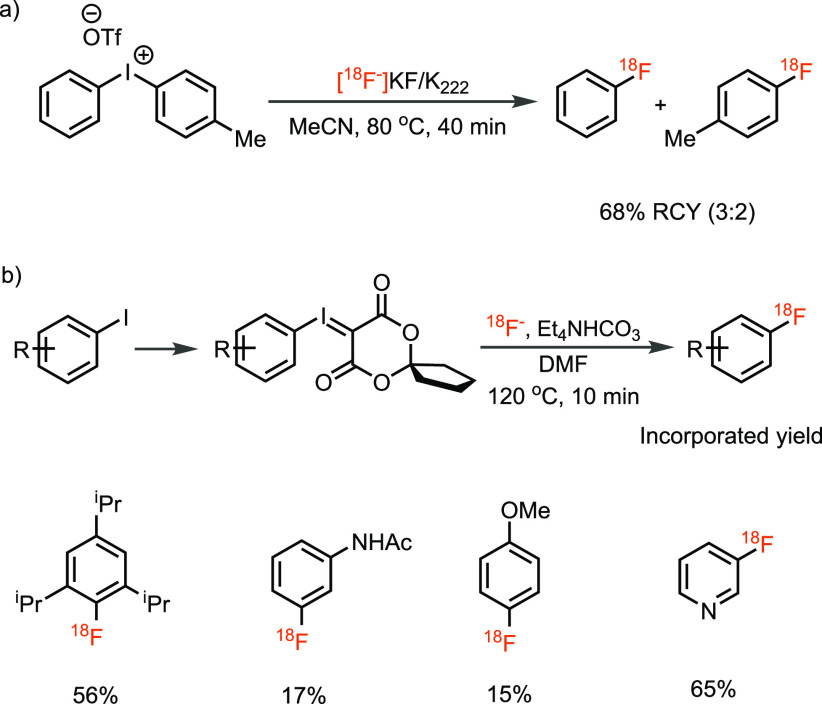
Radiofluorination of Hypervalent Iodine(III) Compounds with [^18^F]Fluoride Using (a) Diaryliodonium Salts and (b) Spirocyclic Iodonium Ylides (SCIDY)

Fluorination methods without transition metals have been used for a long time.^24^ On an industrial scale, fluoride is most commonly introduced to arenes via nucleophilic aromatic substitution (S_N_Ar) due to the operational simplicity of these reactions.^25^ In S_N_Ar reactions, the requirement of an activating group (−NO_2_, −CN, −CF_3_, −CO– etc.) in the *ortho* or *para* position to the leaving group limits the substrate scope to only electron-deficient arenes, although the reaction is readily translated to fluorine-18 for simple, electron-poor arenes. The shortage of methods to label arenes without bearing appropriately positioned electron-withdrawing groups was approached by Pike et al., who developed a single-step radiofluorination of diaryliodonium salts by n.c.a. [^18^F]fluoride (Scheme 3a).^26^ Later, the method was modified by others.^27−31^ For example, Liang and Vasdev designed novel spirocyclic iodonium ylides which are more stable toward decomposition and disproportionation reactions of hypervalent iodine precursors during radiolabeling (Scheme 3b).^32,33^ One pitfall of the method is that, currently, strong Lewis or Brønsted acids are used to synthesize the diaryliodonium salts. In general, late-stage synthesis of hypervalent iodine compounds is challenging, and better methods are needed for their synthesis to increase the utility of aryliodonium salts to develop PET tracers.^34^

**Scheme 4:**
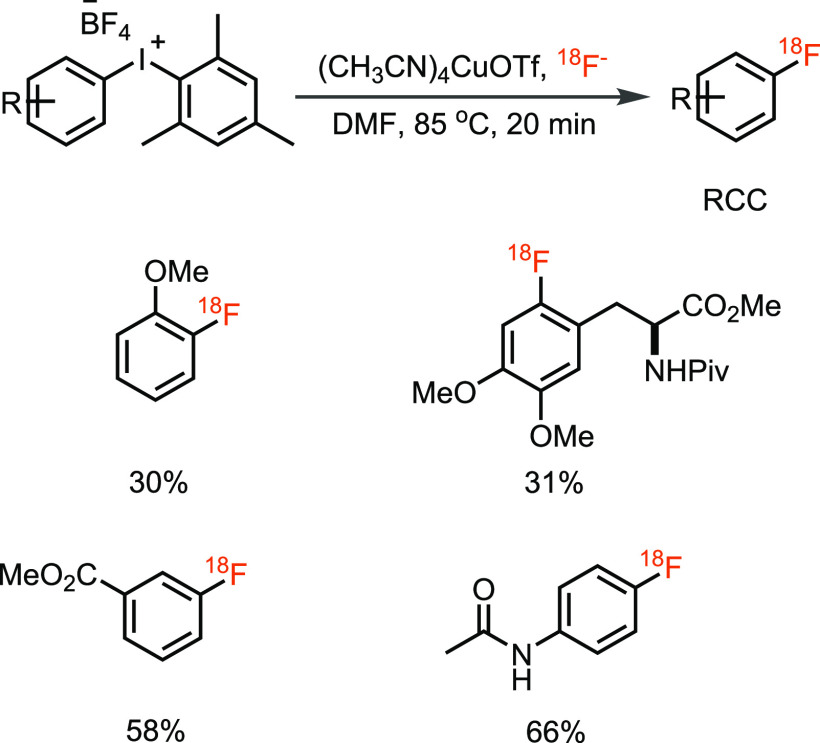
Copper-Catalyzed ^18^F-Fluorination of (Mesityl)(aryl)iodonium Salts

If transition metals can be avoided to develop a radiofluorination reaction, they should be, but if one can access molecules practically that cannot otherwise be accessed then their use may be justified. Copper catalysts, for example, can improve the reactivity of diaryliodonium salts with [^18^F]fluoride. Previously, the transformation required high temperature, and the regioselectivity depended highly on the electronic properties of the two arenes attached to iodine resulting in a mixture of products with low RCY.^26^ In 2014, Sanford and Scott demonstrated a copper-catalyzed radiofluorination of (mesityl)(aryl)iodonium salts using [^18^F]KF (Scheme 4).^35^ The reaction is highly regioselective because the mesityl group always directs radiofluorination to the other aryl groups on iodine, irrespective of the electronic properties, and also radiofluorination of electron-poor, neutral, and rich arenes occurred. The (mesityl)(aryl)iodonium salts used in the substrate scope were prepared from the corresponding aryl boronic acids that themselves can function as starting materials for aryl fluoride synthesis as demonstrated by the Gouverneur group in 2014.

**Scheme 5:**
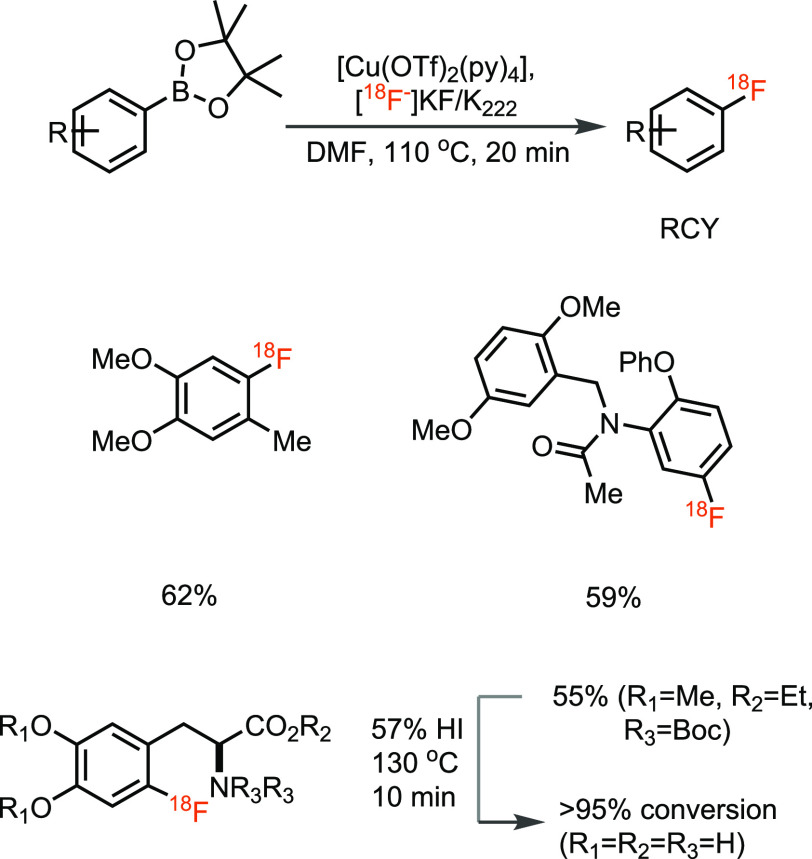
Copper-Mediated Nucleophilic ^18^F-Fluorination of Aryl Boronic Esters

**Scheme 6:**
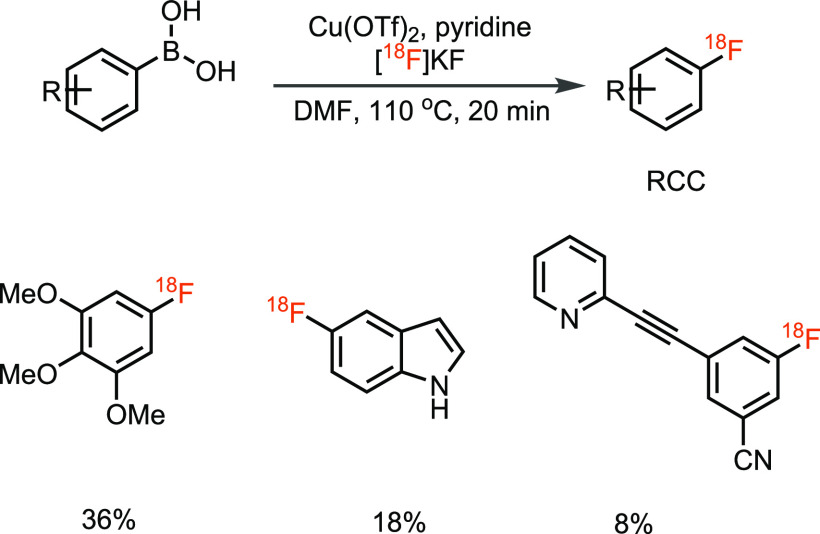
Copper-Mediated Nucleophilic ^18^F-Fluorination of Aryl Boronic Acids

The Gouverneur group developed a copper-mediated radiofluorination reaction of an extensive range of pinacol-derived aryl boronic esters (Scheme 5).^36^ The method is suitable for a wide variety of substrates, does not require the preparation of complex labeling precursors, and can be performed in an open-to-air reaction vessel. In the subsequent years, many clinically relevant PET radiotracers were prepared using this method.^37,38^ Within the modern fluorination methods developed over the recent past, this method is clearly one of the most powerful developments; it substantially expands the available substrate space compared to conventional methods and is relatively straightforward and practical to implement. However, the method has experienced some challenges in automation of the process as the reaction is sensitive to base and also requires oxygen, which complicates handling with the inert gas push systems used in modern automation modules for radiochemistry.^39^ Sanford and Scott developed radiofluorination of other organoboron precursors, i.e., aryl and vinyl boronic acids with Cu(OTf)_2_ (Scheme 6).^39^ Unlike in the conventional S_N_Ar reactions with [^18^F]fluoride and heat, the copper-mediated radiofluorination of organoboranes has resulted in a major progression of the field and has opened access to C–^18^F bonds that were not readily accessible before.

**Scheme 7:**
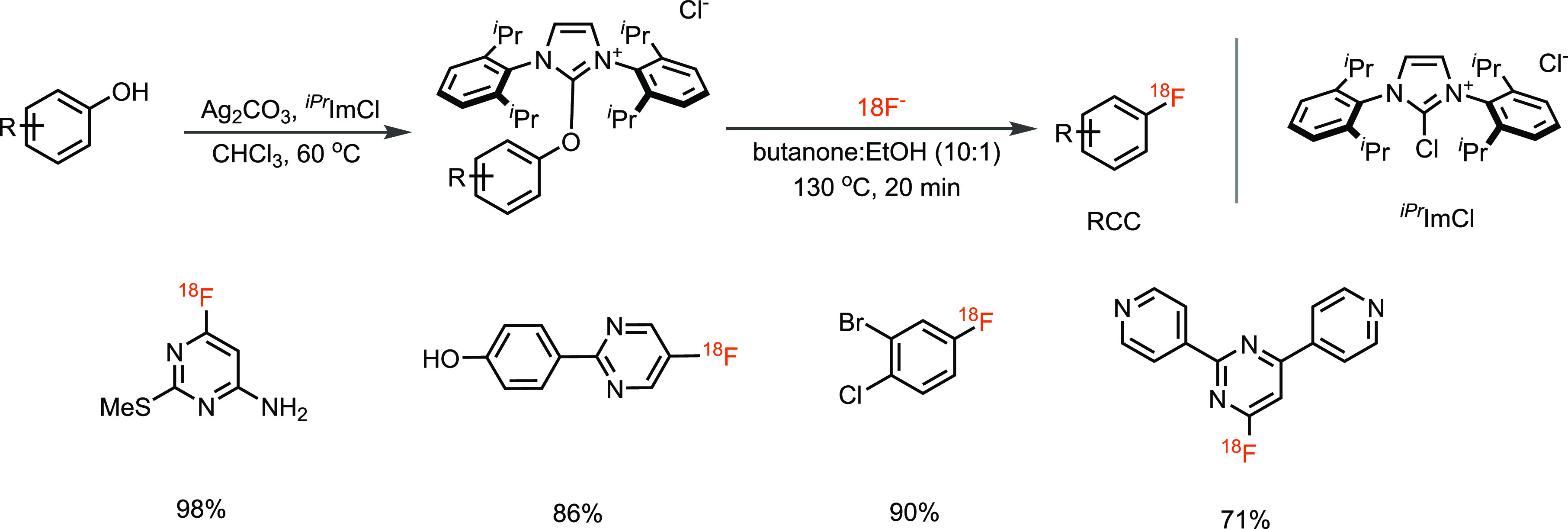
^*iPr*^ImCl-Mediated Deoxyfluorination of Phenols with Fluorine-18

Several groups, including Sanford’s, Scott’s, Gouverneur’s, and ours alike, have made use of the redox activity of transition metals that can lower the barrier for C–F bond formation from high-valent transition metals such as Cu(III) or Pd(IV). While still promising and in our opinion worthwhile to further pursue and develop, there is an obvious argument for methods that do not require a transition metal. In 2016, our lab approached the nucleophilic aromatic displacement reaction with fluorine-18 differently, through deoxyfluorination of readily available phenols using chloroimidazolium chloride (^*iPr*^ImCl) or the PhenoFluor reagent^40−45^ via concerted nucleophilic aromatic substitution (CS_N_Ar) reactions (Scheme 7).^46^ Although, the reaction has several advantages over conventional S_N_Ar reactions like chemoselectivity, moisture, and air tolerance, as well as functional group tolerance, phenols containing electron-donating groups undergo radiofluorination with low radiochemical yield (RCY). Here, we saw a substantial deviation from the fluorine-19 chemistry, which functioned fine with electron-rich phenols.^40−42^ Furthermore, the labeling precursor must be made from the phenol, which is an additional step that can fail. Yet, the reaction is robust and reliable, does not require the addition of any transition metals, is readily translated to fluorine-18 for electron-poor phenols, and can be easily set up by radiotechnicians. Compared to our earlier methods, several other researchers had no trouble implementing this method in their radiolabs. Although the labeling precursor must be made from a phenol in a separate step, the reaction to introduce the fluorine itself is straightforward and reliable. Therefore, the transformation does, in our opinion, fulfill the requirements for the synthesis of ^18^F-labeled drugs. A single compound can be prepared, stored, or shipped and directly used with [^18^F]fluoride in the radiochemistry facility with high chances of success for electron-poor arenes. However, a high-throughput use via this method would be complicated due to the requirement of making each labeling precursor from the phenol.

**Scheme 8:**
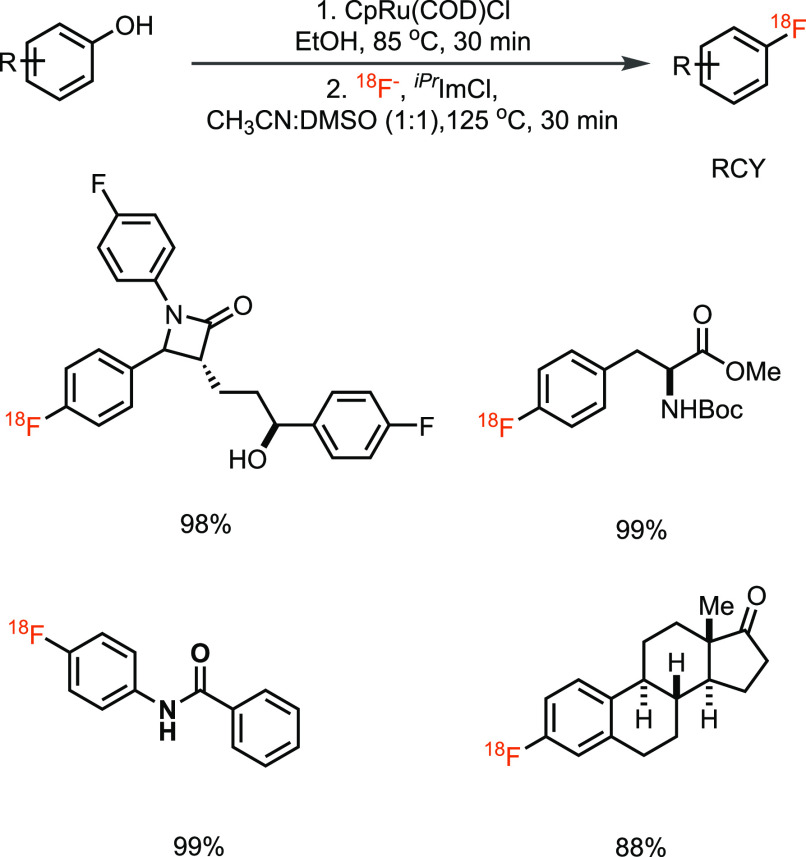
^*iPr*^ImCl-Mediated Deoxyfluorination of Phenols via Ruthenium η^6^-Phenol Complex

**Scheme 9:**
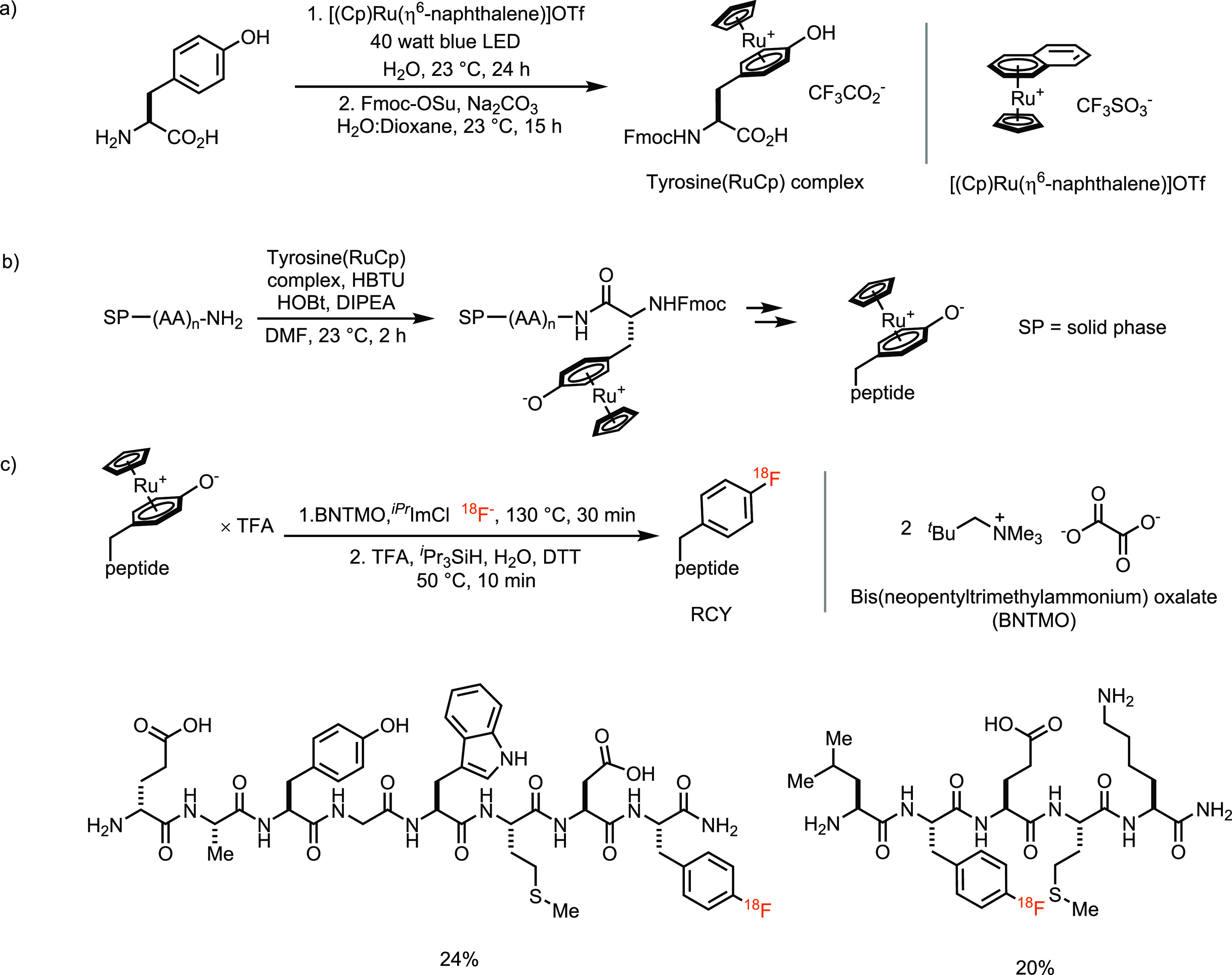
(a) Synthesis and (b) Application of Tyrosine(RuCp) Complex in SPPS Followed by (c) ^*iPr*^ImCl -Mediated Deoxyfluorination of Peptides via Ruthenium η^6^-Phenol Complex

We addressed the shortcoming of substrate scope of the fluorine-18 deoxyfluorination, again through the use of transition-metal chemistry in 2017, which expanded the radiodeoxyfluorination to electron-rich phenols (Scheme 8), inaccessible by the previously reported method,^46^ albeit at the expense of an additional step to introduce the metal. It was for the first time that arenes were activated through η^6^-coordination to ruthenium to facilitate labeling with fluorine-18.^47^ The transformation tolerates moisture and air, has large substrate scope, and can be easily automated. However, the synthesis of the labeling precursor ruthenium η^6^-phenol complex is sometimes difficult and not trivial for nonchemist professionals. On the other hand, such organometallics, once made, are easy to handle. For example, the tyrosine(RuCp) complex (Scheme 9a) is commercially available, can participate in conventional solid-phase peptide synthesis (SPPS) (Scheme 9b), and allows the nonexpert to buy only the compound that enabled the synthesis of ^18^F-labeled peptides (Scheme 9c).^48^ Access to a fluorophenylalanine residue within a complex peptide with fluorine-18 allows for a reliable synthesis of this one residue. If a peptide with fluorine-18 in such a position is desired, the method is a promising solution but most other molecules are out of scope for such an approach with a commercially available ruthenium precursor.

To address the need for robust reaction chemistry that can be employed quickly and reliably, for a large variety of molecules, without the need for a specific functional group already present in the molecule, we sought to develop a fluorination through C–H functionalization. In principle, every molecule should be suitable to serve as starting material for such a process. The group of Groves and Hooker has successfully developed a C–H fluorination method for benzylic C–H bonds^49^ based on Grove’s seminal work in C–H functionalization chemistry.^50,51^ We were interested in addressing aryl fluoride synthesis due to the more common motif and higher stability when compared to their benzyl counterparts. An initial attempt to functionalize arenes through direct C–F bond formation with a new palladium catalyst resulted in a reaction that is too complicated to translate to fluorine-18 and requires electrophilic fluorination sources.^52^ However, we successfully developed a two-step process through the intermediacy of aryl sulfonium salts.

**Scheme 10:**
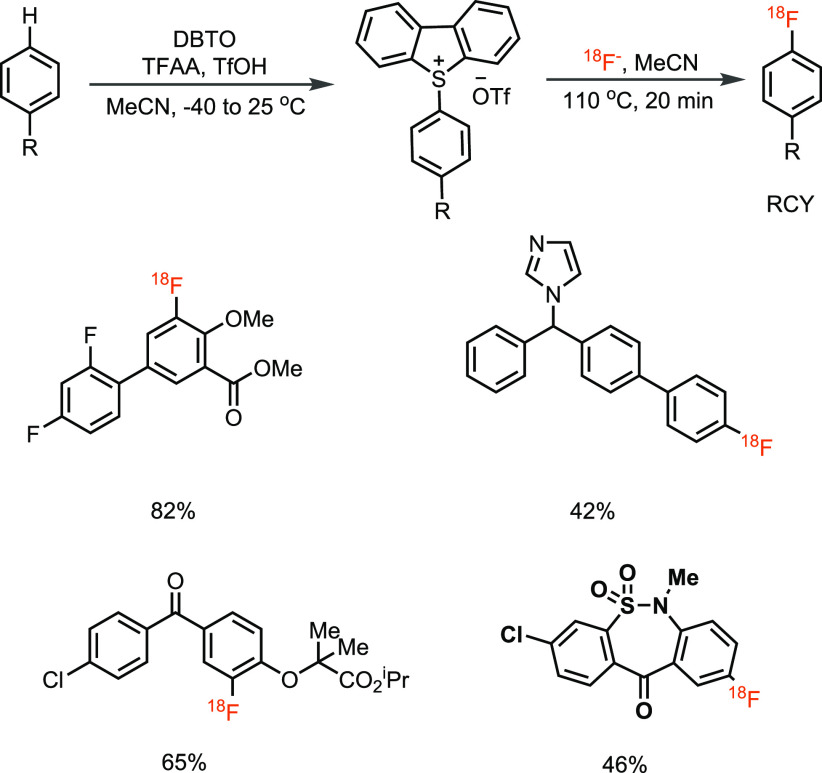
Aromatic C–H ^18^F-Fluorination via Aryl Dibenzothiophenium Salt

Over the past couple of years, our group has introduced a highly regioselective arene C–H functionalization to access aryl thianthrenium salts; the thianthrene substituent serves as a versatile linchpin for further manipulation,^53−59^ including C–F bond formation.^60^ The most practical reaction with promise for fluorine-18 translation was obtained when we switched from the thianthrene scaffold to the dibenzothiophene scaffold.^61^ The substituent can be readily introduced into a variety of molecules through C–H functionalization, although not quite as generally and selectively as thianthrene, and the resulting aryl sulfonium salts react with simple [^18^F]fluoride (Scheme 10), presumably by reductive elimination from a triarylfluoro sulfur(IV) intermediate. The approach can quickly access a variety of suitable reaction precursors, does not require a transition metal, is simple in its execution, and employs a simple fluoride source for the C–F bond formation. The biggest drawback at this stage is the high reaction temperature that is required for the rather slow nucleophilic displacement. An additional drawback of the method is the dependence of the dibenzothiophenylation reaction on the electron density of the arene. The sulfonium salt can only be made for a subset of molecules that exhibit the correct electron density, and hence molecules outside that window cannot be radiofluorinated. Improvement of the chemistry would be to develop a reagent that can form the sulfonium salt selectively in any arene moiety that can then undergo radiofluorination quickly at a temperature of 80 °C or less.

**Scheme 11:**
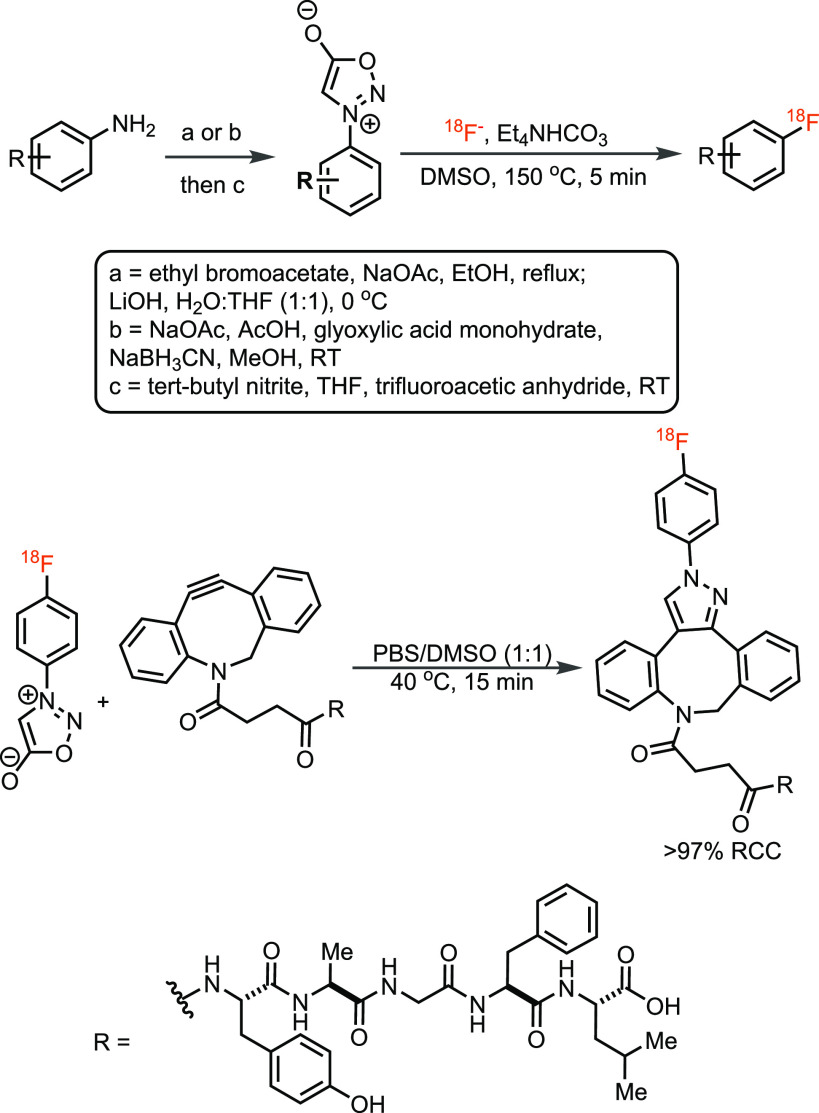
^18^F-Fluorination of Anilines via *N*-Arylsydnones

In 2017, the Murphy group developed the first method to radiofluorinate anilines by converting them into *N*-arylsydnones, followed by nucleophilic attack by [^18^F]fluoride (Scheme 11).^62^ The transformation is operationally simple and can be automated but can only be used for arenes bearing electron-withdrawing groups.

In addition to introduction of fluorine-18 by C–F bond formation, several groups have started focusing on developing methods to form heteroatom–F bonds in pursuit of novel radiofluorination reactions. Fluorine–heteroatom bonds can often be made more readily than common for the classic nucleophilic substitution reactions that typically require harsh reaction conditions (basic conditions and high temperature). Such harsh methods are not suitable for labeling many biomolecules like proteins with [^18^F]fluoride directly. Several members of group 13–15 elements bind readily to fluorine and require milder conditions to promote their formation.^63^ As a result, many advancements have been made in recent years to develop radiofluorination methodologies,^64^ specifically for B–^18^F, Si–^18^F, and Al–^18^F bonds.^65−67^ The valuable trait of these reactions is that they can often be performed in aqueous media and are useful for the radiofluorination of sensitive substrates. However, many small organic molecules, having low hydrolytic stability, are often out of reach for such methods.

**Scheme 12:**
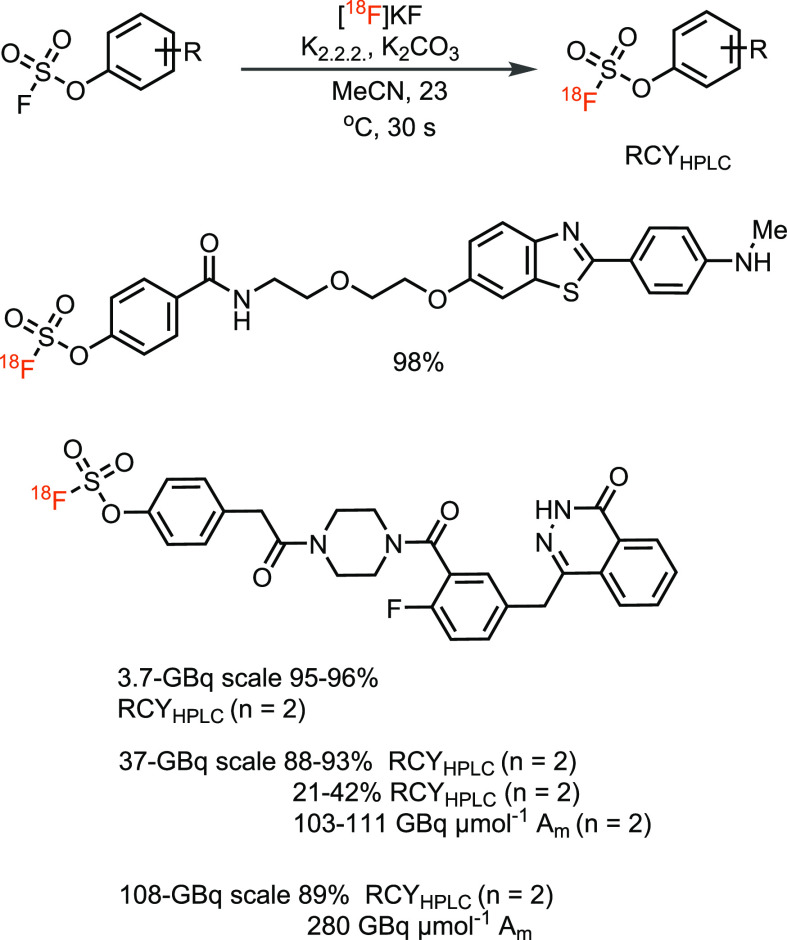
^18^F-Fluorination via Sulfur Fluoride Exchange (SuFEx) between Phenyl Fluorosulfate and [^18^F]Fluoride

Furthermore, ^18^F/^19^F isotope exchange reactions can be practical, for example, to obtain heteroatom–^18^F bonds. Exchange reactions always face the potential challenge of low molar activity (*A*_m_) due to dilution from exogenous fluorine-19, as in a carrier-added case. However, if small amounts of starting material are used, high molar activities can be obtained, especially when more than one fluorine is present.^68,69^ Recently, the Sharpless group has developed a fast radiosynthesis process to prepare aryl [^18^F]fluorosulfates via sulfur(VI) fluoride exchange (SuFEx) between phenyl fluorosulfate and [^18^F]fluoride and demonstrated the first PET imaging application of S–^18^F-based probes (Scheme 12).^70^

## Conclusion

It is too early for modern chemical fluorination methods to have substantially impacted human PET imaging. However, it is not unlikely that they will have an impact in the future as the best and most practical methods and those that are still to come will be implemented in radiopharmacies. Implementation of new chemistry for the synthesis of PET tracers is slow, but that timeline is expected. Better chemistry will, ultimately, have an impact on the development and the synthesis of new PET radiotracers because a wider chemical space will be accessible. The guiding principles we need to keep in mind as chemists dictate that the chemistry not only needs to provide solutions to access molecules that are not readily accessible by conventional, simple methods but also be sufficiently practical and translatable that the advance does not stop at a pure chemical contribution but be ultimately used by radiopharmacy practitioners. We provide here a few guiding principles that we deem appropriate to consider for new chemistry developments so that they have the highest chance for ultimate success for translation.

## [^18^F]Fluoride vs [^18^F]F_2_ Gas

Fluorine gas is a strong electrophilic fluorinating reagent that is cumbersome to handle and, in addition, more complicated to make in cyclotrons. On the other hand, [^18^F]fluoride is formed in an aqueous solution from a cyclotron and much more practical and easy to use. It is our opinion that the use of fluoride as opposed to fluorine gas is almost always more desirable for broader impact in PET.

## Carrier-Added (c.a.) vs Noncarrier-Added (n.c.a.)

The key differences between c.a. and n.c.a. are molar activity (*A*_m_) and mass. High *A*_m_ is desired for tracers designated for low abundance receptors, injecting lower mass, which is desirable when there is potential toxicity, and shipment from radiopharmacy to imaging centers.^71^ However, for some applications high *A*_m_ is not required, for example, for some metabolic tracers such as [^18^F]FDG.^72,73^ Therefore, the choice of the production method (c.a. or n.c.a.) is dependent on the type of PET tracer. No carrier added reactions are generally preferred because they are also useful for applications that do not require high *A*_m_, whereas the reverse is not true. Sometimes, as in isotope exchange reactions, other advantages come into play, so that reactions with fluoride present in the starting material can also be useful.

## Isotopic Exchange

^18^F/^19^F isotope exchange has proven to be a very useful method that allows the labeling of molecules that contain exchangeable fluorine without modifying their molecular structure. In addition, the synthesis is typically easy and simple because the starting material is virtually identical to the product, except it is a different isotopologue as long as the fluorine-containing functional group for fluorine exchange is suitable for the target.

## Requirement for High RCY

Radiochemical yield (RCY), which is a function of both chemical yield and synthesis/purification time, is a measure of efficiency of the radiolabeling process.^74^ Although high RCY is always desirable, it is not always essential for radiochemists to have RCY that are high.^2^ Often, RCYs are even reported in amount of activity obtained at the end of the synthesis, as opposed to in percent, as organic chemists are used to. Such a denotation signifies the need to make a product in a certain amount and purity, as opposed to high chemical yield.

## Reaction Time

The race against time is a difficult aspect of radiochemistry. The duration between the end of bombardment (EOB) and the injection of the tracer should be shorter than three isotope-half-lives as a general rule.^2^ But the fluorine attachment chemistry is just one step in the overall process that includes preparation of the fluoride such as drying after cyclotron synthesis as well as purification and reformulation. For chemists to develop fluorination reactions, we recommend a synthesis-only time of 30 min or less. Faster is never worse but has little impact due to the other steps before and after reaction chemistry that are not impacted by reaction time. Much more relevant is the development of functional group tolerant reactions so that the fluorine incorporation can occur in the ultimate step. Every subsequent step takes time and effort, and every transfer, purification, or even pumping of the reaction mixture lowers the yield.

## Heat vs No Heat

High reaction temperature required for overcoming the barrier to form C–^18^F bond (for example, boiling point of acetonitrile) is generally not problematic if the molecule tolerates the heat, which is often the case unless acidic, basic, or oxidative conditions are used. In the best case, the reaction temperature should not be higher than the boiling point of the solvent, although some synthesis platforms will even tolerate pressure. Given that acetonitrile is a convenient solvent, reaction temperature from room temperature to 80 °C is a convenient temperature window.

## Solvent

The solvent is important in as much that it must solubilize the fluoride used. In addition, several solvents should be avoided, such as dichloroethane, because they are toxic and would require extensive safety profiling of the final product to be injected. Solvents of low toxicity are generally preferred.

## Water vs No Water

A moisture-tolerant radiofluorination reaction is desirable because, generally, it is an indication that the reaction will tolerate a range of functional groups. Anhydrous fluoride is highly basic and will not be compatible with a variety of functional groups. The more water tolerant, the better, although other solvents, such as *tert*-butyl alcohol, also attenuate the basicity of fluoride, so that in principle anhydrous conditions can still be functional group tolerant and useful for radiosynthesis. Given that [^18^F]fluoride is made from heavy water, there is always water around, and the requirement for extensive drying is cumbersome, time-intensive, and results in highly basic fluoride.

## Regioselectivity and Functional Group Tolerance

High regioselectivity of a radiosynthetic conversion is always desired in order to avoid time-consuming purifications, which also results in lower *A*_m_ of product. Furthermore, we need chemistry that is robust, with readily available starting material, tolerating a wide range of functional groups. Nonetheless, a practical and highly reliable reaction for conversion of one functional group is also looked for to synthesize target-specific radiolabeled drug candidates.

## Transition Metal: Helpful or Not

Ideally, transition metal is not required in radiochemistry due to the purification step before reformulation and injection into the body. Nevertheless, the ability to access otherwise inaccessible labeled molecules often justifies the use of transition metals.

## Supporting Techniques like Microfluidics

Microfluidic devices are an excellent technique developed by miniaturizing the radiochemistry instrumentation in order to address challenges regarding the high cost of PET tracer development and to be able to change stoichiometry due to miniaturization. By allowing rapid mixing and efficient heat transfer, small dimensions of a microfluidic device ensure improved control over reaction conditions and, therefore, higher yields.^71^ Some additional advantages include safe operation, reagent amount minimization, and high *A*_m_ of the products. However, volume reduction can give rise to a limited droplet lifetime as it may quickly evaporate without carrying out the fluorination reaction.

## Fluorine-19 Reaction Development: Useful or Not

The development of fluorine-19 chemistry is not always advisable due to challenges related to its translation. While we have found it instructive to develop new reaction pathways with fluorine-19 first, due to a much higher throughput in experimentation, once a hit is obtained, development of fluorine-18 chemistry, as opposed to optimization with fluorine-19, is often better due to the challenge of translation.

## Automation: Required or Not

One of the challenges in radiochemistry is that the amount of activity required for human imaging is not appropriate for manual processing. Therefore, it is essential that the labeling procedure can be performed in a protected hot cell by an automated radiochemical synthesizer for easy translation to routine clinical use.

We hope that this personal perspective can function as a guiding principle for organic chemists who may be interested in contributing to the field of fluorine-18 chemistry. To us, the field is exciting because, ideally, advances in fundamental chemistry can have an impact on human health.
